# The Impact of Different Equilibration Methods on the Cryopreservation Effectiveness of Boar Semen

**DOI:** 10.1002/vms3.70888

**Published:** 2026-03-17

**Authors:** Biyu Zhang, Haidong Liu, Fuqiang Chang, Shouqian Sang, Jing Li, Wenchao Li, Chongmei Ruan, Youfang Gu

**Affiliations:** ^1^ College of Animal Science Anhui Science and Technology University Chuzhou China; ^2^ Anhui Province Key Laboratory of Animal Nutritional Regulation and Health Chuzhou China; ^3^ Anhui Engineering Technology Research Center of Pork Quality Control and Enhance Chuzhou China

**Keywords:** boar, one‐step equilibrium method, semen cryopreservation, semen quality, two‐step equilibrium method

## Abstract

Boar semen cryopreservation is crucial for the porcine industry. The two‐step (S2) is currently used for cryopreservation of boar semen. In this method, a cryoprotectant is added at 4°C to reduce its toxicity. Another method is the one‐step (S1), which is easier, cheaper and reduces the need for equipment, but may increase the toxicity of cryoprotectant. This study compared S1 and S2 equilibration protocols, evaluating post‐thaw total motility, viability, motion parameters, membrane integrity, antioxidant indicators, apoptosis and related gene expression. Results showed S2 had higher motility (*p* < 0.05), while S1 had greater average path velocity (*p* < 0.05), better plasma membrane integrity (*p* < 0.05), lower malondialdehyde (MDA) (*p* < 0.01), higher total antioxidant capacity (T‐AOC) and catalase (CAT) (*p* < 0.01), lower superoxide dismutase (SOD) (*p* < 0.05) and reduced apoptosis (*p* < 0.05). S2 had higher Caspase‐9, Bax and SOD‐2 expression (*p* < 0.05). S1 shows superiority in membrane protection, antioxidant capacity and anti‐apoptosis, despite slightly lower motility. It is a promising method for large‐scale use with further refinement to enhance post‐thaw motility.

## Graphical abstract

This study compared the effects of one‐step and two‐step balanced treatment methods for cryopreservation of boar semen and evaluated their impact on sperm quality after thawing. The optimized balanced treatment method improved sperm integrity and antioxidant capacity, and reduced apoptosis and oxidative damage. These findings provide practical guidance for improving the efficiency of boar semen cryopreservation and reproductive performance.

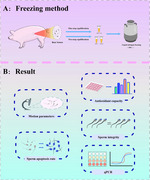

## 1 Introduction

Semen preservation is a key aspect of artificial insemination technology in pigs (Bailey et al. 2008). The primary methods for semen preservation include ambient temperature storage, low‐temperature storage and cryopreservation, each presenting unique advantages and limitations (Sharafi et al. 2022). Among these, cryopreservation entails the dilution, cooling, equilibration and cryopreservation of semen, followed by its long‐term storage in liquid nitrogen (Rodriguez‐Martinez and Wallgren 2011). This method effectively halts the metabolic activity of sperm, enabling them to recover motility and fertilization capacity upon thawing, theoretically allowing for indefinite preservation (Kong et al. 2012). Consequently, cryopreservation plays a pivotal role in conserving genetic resources, facilitating cross‐regional genetic exchange, reducing the maintenance of breeding males and enhancing reproductive efficiency (Zhang et al. 2024). Despite offering numerous advantages, cryopreservation technology faces several challenges, including diminished semen quality, reduced fertilization rates and decreased litter sizes. These limitations restrict its utilization to less than 1% of artificial insemination applications. Consequently, the development and investigation of simplified, highly efficient cryopreservation techniques are paramount for the swine industry.

The prevailing method for boar semen cryopreservation is the two‐step (S2) equilibration protocol, characterized by the gradual, sequential addition of cryoprotectants in two stages to facilitate the gentle osmotic balance across the sperm plasma membrane. However, this method is plagued by high costs, prolonged processing time and a necessity for specialized equipment and highly trained personnel (Galarza Lucero et al. 2019). In 2019, the one‐step (S1) equilibration protocol was subsequently introduced as a streamlined alternative. Unlike the S2 method's sequential, gradual cryoprotectant addition, the S1 protocol involves adding the total volume of cryoprotectants simultaneously to the semen. This procedural simplification results in shorter handling time, reduced risk of contamination and improved cryopreservation efficiency (Shepherd and Herickhoff 2022). According to reports, the S2 equilibration method for semen cryopreservation requires longer operation time and multiple transfers, increasing operational complexity and the risk of cellular damage (Monteiro et al. 2022). Furthermore, prolonged processing time and extended exposure to varying cryoprotectant concentrations may exacerbate oxidative stress, leading to DNA damage and other functional impairments. Boar sperm are particularly susceptible to cryoinjury, primarily attributed to their comparatively large sperm heads and the low cholesterol content within their plasma membranes (Pini et al. 2016). Simultaneously, the production of reactive oxygen species (ROS) by mitochondria during cryopreservation is also a significant contributor to DNA damage (Koppers et al. 2010). Therefore, the development of simpler, more cost‐effective and highly efficient cryopreservation protocols is essential to fully realize the potential of this technology.

Currently, there is a notable paucity of research comparing the effects of two equilibration protocols on the cryopreservation outcomes of pig semen. Thus, this study aims to evaluate the performance of different equilibration protocols in cryopreserving pig sperm, with the objective of providing a theoretical foundation for optimizing cryopreservation systems for pig semen.

## 2 Materials and Methods

### 2.1 Experimental Animals

Six healthy 2‐year‐old Large White boars (obtained from Fengyang Family Farm, China) were selected for this study. All animals were approved for use in this research under ethical guidelines.

### 2.2 Reagents and Instrumentation

Microplate reader (Multiskan, Thermo, USA), Computer‐Assisted Sperm Analysis (CASA) (ML‐608JZ, Songjing Tianlun, China), nucleic acid and protein analyser (NanoDrop One, Thermo, USA), real‐time PCR system (FQD‐96A, Bioer, China), fluorescence microscope (BX63, Olympus, Japan), phase‐contrast microscope (AE2000, Motic, China) and refrigerated centrifuge (Rotina 380R, Hettich, Germany) were used in the study.

Tris, glucose, citric acid and glycerol were purchased from Sangon Biotech (Shanghai, China). Detection kits for total antioxidant capacity (T‐AOC) (A015‐2‐1), catalase (CAT) (A007‐1‐1), superoxide dismutase (SOD) (A001‐3) and malondialdehyde (MDA) (A003‐1) were purchased from Nanjing Jiancheng Bioengineering Institute. Coomassie Brilliant Blue G‐250 (GC307007, Seville, China), Hoechst 33342 solution (KGA1805‐10, KeyGEN BioTECH, Jiangsu, China), reverse transcription kit (AG11728, Aikerui, China) and sperm viability test kit (Hypotonic Swelling Method, LEAGENE, DA0186) were obtained.

Cryopreservation basal solution: Note that 1.1 g of glucose, 1.48 g of citric acid, 2.42 g of tris (tris(hydroxymethyl)aminomethane), 0.23 g of EDTA (ethylenediaminetetraacetic acid) and 1.0 g of sodium bicarbonate were dissolved in double‐distilled water and adjusted to a final volume of 100 mL.

Thawing solution: A total of 0.125 g of EDTA, 0.6 g of citric acid, 0.125 g of sodium bicarbonate and 3.7 g of glucose were dissolved in double‐distilled water and brought to a final volume of 100 mL.

Cryoprotective Solution I: Fresh egg yolk (20 mL) was added to the basic freezing extender to a final volume of 100 mL and thoroughly mixed; the final egg yolk concentration was 20%.

Cryoprotective Solution II: Fresh egg yolk (20 mL) and glycerol (6 mL) were mixed, and the basic freezing extender was added to reach a final volume of 100 mL. The concentrations of glycerol and egg yolk were 6% and 20%, respectively. The mixture was homogenized and stored at 4°C until use (Monteiro et al. 2022) (Table 1).

**TABLE 1:** Formulas for diluents of cryopreservation basal medium and resuscitation fluid.

	Cryopreservation basal medium					Thawing solution			
Ingredients	Glucose	Citric acid	Tris	EDTA	NaHCO_3_	EDTA	Sodium citrate	NaHCO_3_	Glucose
Dosage	1.1 g	1.48 g	2.42 g	0.23 g	1 g	0.125 g	0.6 g	0.125 g	3.7 g

*Note*: The above reagent is diluted to a constant volume of 100 mL with distilled water. The diluent follows the principle of being prepared and used immediately.

After mixing cryoprotectant Solution I and II in a 1:1 ratio, the final egg yolk concentration is 3%.

### 2.3 Semen Collection

Semen samples were collected from six Large White boars using the manual collection method (Bishop et al. 1999). The collection frequency was set at three times per week, with a minimum interval of 48 h between consecutive collections, resulting in a total of 36 ejaculates. Gel‐like substances, cellular debris and impurities were removed from the semen using four‐layer sterile gauze, retaining only the sperm‐rich mid and posterior segments (Yeste 2016). The selected fractions were collected into sterile semen collection cups under aseptic conditions. All collected semen samples were thoroughly mixed, one sample was used as a fresh semen control and the remainder was used for freezing in both S1 and S2 equilibration methods. The semen met the criteria of ≥ 80% motility and an abnormality rate of < 20%. Then, the cryopreservation base solution was diluted at a ratio of 1:5 for subsequent experiments.

### 2.4 Sperm Freezing and Thawing

S1: The semen was centrifuged at 1500 rpm for 3 min to remove the cryopreservation base solution. After centrifugation, the semen was resuspended in a 1:1 (v/v) mixture of cryoprotective Solution I and cryoprotective Solution II, resulting in a final glycerol concentration of 3%. The semen was then aliquoted into 0.5 mL cryogenic straws using a negative pressure filling method and sealed with polyvinyl alcohol (PVA) powder. The straws were placed horizontally on a self‐made foam floating rack (3 cm thick) and equilibrated at 4°C for 2 h to allow slow cooling. Subsequently, the floating rack was exposed to liquid nitrogen vapour for 10 min, after which the straws were immediately plunged into liquid nitrogen for long‐term storage (Monteiro et al. 2022).

S2: The semen was centrifuged at 1500 rpm for 3 min to remove the cryopreservation base solution. After centrifugation, the semen was mixed with cryoprotective Solution I and placed in a 4°C refrigerator for slow cooling and equilibration for 2 h. Subsequently, cryoprotective Solution II was added at a 1:1 (v/v) ratio, and the mixture was thoroughly mixed by pipetting until the final glycerol concentration in the semen was 3%. The sample was then equilibrated at 4°C for another 2 h. The semen was then aliquoted into 0.5 mL cryogenic straws using a negative pressure filling method and sealed with PVA powder. The straws were placed horizontally on a self‐made foam floating rack positioned 3 cm above the surface of liquid nitrogen for vapour‐phase exposure for 10 min, and then immediately plunged into liquid nitrogen for long‐term storage (Monteiro et al. 2022).

For thawing, the straws were removed from liquid nitrogen and immediately immersed in a 38°C water bath for 30 s. After thawing, the semen was mixed with the thawing solution and incubated at 38°C for 8 min prior to subsequent evaluations.

### 2.5 Evaluation of Sperm Quality

#### 2.5.1 Sperm Total Motility, Viability and Motion Parameters

Four 0.5 mL freezing straws were thawed, and 10 µL of the semen was mixed with 90 µL of revival medium and incubated at 37°C for 2 min. The post‐thaw sperm total motility, viability, straight‐line velocity (VSL), curvilinear velocity (VCL), average path velocity (VAP), linearity, straightness and wobble were assessed using a CASA system. Each of the five randomly selected fields of view observed contained at least 200 sperm. The software settings used for the CASA system were those recommended by the manufacturer for the analysis of boar spermatozoa: frame required 45, frame rate 60 Hz, minimum cell contrast 46, minimum cell size 7 pixels, straightness threshold 45%, VAP (micrometres per second) threshold 45 µm/s, low VAP cut‐off 20 µm/s, low VSL (micrometres per second) cut‐off 5.0 µm/s, static intensity gates 0.50–2.50, static size gates 0.65–4.90 and static elongation 0–87.

#### 2.5.2 Sperm Acrosome Integrity

Assessment of sperm acrosome integrity was carried out using Coomassie Brilliant Blue G‐250 staining according to the method of Xuan et al. (2020). The detailed operating steps are as follows. Frozen semen straws were thawed and centrifuged to discard the supernatant. The sperm pellet was resuspended in phosphate‐buffered saline (PBS). Then, 10 µL of the sperm suspension was smeared onto a glass slide and air‐dried. The dried smears were stained with Coomassie Brilliant Blue G‐250 at room temperature for 20–30 min, followed by gentle rinsing with distilled water and air‐drying. Slides were observed under a microscope at 400× magnification. At least five random fields per slide were examined, with a minimum of 200 sperm counted per field to calculate the percentage of sperm with intact acrosomes. Sperm with intact acrosomes exhibited a smooth, oval‐shaped blue cap, whereas those with damaged acrosomes showed irregular or deficient blue staining in the acrosomal region.

#### 2.5.3 Sperm Membrane Integrity

Assessment of sperm plasma membrane integrity was carried out using a sperm viability test kit. According to the manufacturer's instructions, 0.1 mL of thawed semen was added to 1 mL of hypoosmotic solution and incubated at 37°C for 60 min. Sperm exhibiting curled tails were considered to have intact plasma membranes (Irez et al. 2013). At least five random fields were observed under the microscope, with a minimum of 200 sperm counted per field to calculate the percentage of sperm with intact plasma membranes.

#### 2.5.4 Antioxidant Indices

Following the method of Nelson and McGrady (1981), four freezing straws were thawed, and the post‐thaw sperm was subjected to ultrasonic disruption using an ultrasonic cell breaker (SCIENTZ‐IID, Ganoderma lucidum, China) (250 W, 5 s of sonication followed by 30 s of rest, repeated four times). The assay kit was used to measure oxidative stress markers, including SOD, MDA, T‐AOC and CAT levels. Absorbance was measured using a microplate reader, and the results were calculated using the formulas provided in the kit. Three samples were collected from each group, and the procedure was strictly followed according to the kit instructions.

#### 2.5.5 Sperm Apoptosis Rate

Assessment of sperm apoptosis rate was conducted using Hoechst 33342 staining kit. Frozen semen straws were thawed and centrifuged to discard the supernatant. The sperm pellet was resuspended in 1 mL of Hoechst 33342 working solution and incubated at room temperature in the dark for 5 min. After incubation, 10 µL of the suspension was placed onto a glass slide and observed under a fluorescence microscope (Arshad et al. 2024). The excitation wavelength of Hoechst 33342 was 352 nm, and the emission wavelength was 400–500 nm. At least five fields of view were randomly selected from each sample for observation, and at least 200 sperm were counted in each field. Apoptotic sperm exhibited bright blue fluorescence, and the percentage of apoptotic sperm was calculated accordingly.

### 2.6 Total RNA Extraction

Following the established protocol (Sahoo et al. 2021), RNA was extracted from the sperm. Four 0.5 mL freezing straws were thawed, and the post‐thaw sperm samples were centrifuged. An equivalent volume of Trizol was added to the pellet, followed by thorough pipetting to ensure complete cell lysis. Chloroform (1/5 volume of Trizol) was added, mixed vigorously, and allowed to stand at room temperature. Following centrifugation at 12,000 *g* for 15 min at 4°C, the top aqueous layer was gathered. Isopropanol in the same volume was added, stirred gently and allowed to incubate at room temperature. The mixture was then centrifuged at 12,000 *g* for 10 min at 4°C, and the supernatant was discarded. The RNA pellet underwent a wash with 75% ethanol and was centrifuged at 7500 *g* for 5 min at 4°C. The supernatant was then discarded, and the pellet was left to air‐dry at room temperature for 5 min. The RNA was dissolved in DEPC‐treated water, and the OD values (OD260/280) were measured, with acceptable values ranging from 1.8 to 2.0. The extracted RNA was stored at −80°C for subsequent experiments.

### 2.7 Reverse Transcription and qRT‐PCR

Total RNA was reverse transcribed into cDNA utilizing a reverse transcription kit. With GAPDH as the reference gene, specific primer sequences for boar sperm‐related genes (TNF‐α, Bcl‐2, P53, Caspase‐9, Bax and SOD‐2) were retrieved from the NCBI cDNA database and designed using Primer 5.0 software (Table 2). For the qRT‐PCR, the reaction conditions included an initial denaturation at 95°C for 5 min, then 40 cycles of 10 s denaturation at 95°C and 30 s annealing at 60°C (Chang et al. 2025).

**TABLE 2:** Primer sequence.

Gene	Primer sequence(5’→3’)	Product length
GAPDH	F: CACGATGGTGAAGGTCGGAG	150 bp
	R: TTGACTGTGCCGTGGAACTT	
TNF‐α	F: ATTCAGGGATGTGTGGCCTG	120 bp
	R: CCAGATGTCCCAGGTTGCAT	
Bcl‐2	F: GGCAACCCATCCTGGCACCT	134 bp
	R: AACTCATCGCCCGCCTCCCT	
P53	F: GAACAGCTTTGAGGTGCGTG	175 bp
	R: GCCATCCAGTGGCTTCTTCT	
Caspase‐9	F: AACTTCTGCCATGAGTCGGG	142 bp
	R: CCAAAGCCTGGACCATTTGC	
Bax	F: GCCGAAATGTTTGCTGACGG	146 bp
	R: CGAAGGAAGTCCAGCGTCCA	
SOD‐2	F: CAGGAACAGCCACTACAGTAT	217 bp
	R:TAACCTCCTGGCTCTTTCCA	

### 2.8 Statistical Analysis

Results were expressed as the mean ± SD. Data were analysed using independent samples *t*‐test using SPSS 27.0 software. The 2^‐ΔΔCT^ method was applied for relative gene expression analysis. A *p*‐value less than 0.05 was considered statistically significant (*) and a *p*‐value less than 0.01 was considered highly significant (**).

## 3 Results

### 3.1 Effects of Different Equilibration Methods on Post‐Thaw Sperm Motion Parameters

As illustrated in Table 3, the S1 group exhibited significantly higher values for sperm VCL and VAP compared to the S2 group (*p* < 0.05). Conversely, the sperm total motility, straightness and linearity in the S2 group was significantly higher than that in the S1 group (*p* < 0.05). There were no significant differences in sperm viability, VSL and wobble between S1 and S2 (*p* > 0.05).

**TABLE 3:** The influence of different equilibration methods on post‐thaw motility, viability and motility parameters of boar sperm.

Item	S1	S2	*p*
Total motility (%)	39.94 ± 0.98^a^	42.06 ± 1.03^a^	0.001
Sperm viability (%)	41.78 ± 0.92	41.94 ± 0.87	0.706
VSL (µm/s)	23.82 ± 2.32	22.83 ± 1.38	0.286
VCL (µm/s)	56.52 ± 3.19^a^	48.33 ± 2.77^a^	0.001
VAP (µm/s)	51.14 ± 3.76^a^	44.16 ± 4.28^a^	0.002
LIN (%)	42.08 ± 2.38^a^	47.27 ± 2.43^a^	0.001
STR (%)	46.79 ± 5.52^a^	52.00 ± 4.54^a^	0.044
WOB (%)	90.65 ± 7.38	91.45 ± 8.07	0.830

^a^ Means are significantly different (*p* < 0.05).

### 3.2 Effects of Different Equilibration Methods on Post‐Thaw Sperm Acrosome Integrity and Plasma Membrane Integrity

**FIGURE 1:**
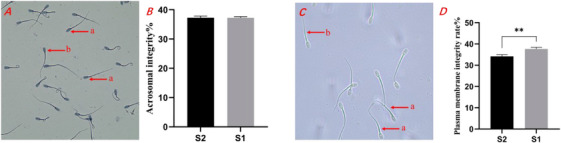
Effects of different equilibration methods on post‐thaw sperm acrosome integrity and plasma membrane integrity.

As shown in Figure 1A, sperm with intact acrosomes exhibited a smooth and undamaged head structure, whereas those with compromised acrosomes displayed head deformities and depressions. The analysis of sperm acrosome integrity (Figure 1B) revealed that the S1 group had a higher level of acrosome integrity compared to the S2 group; however, the difference was not statistically significant (*p* > 0.05). As depicted in Figure 1C, sperm with intact plasma membranes showed a distinctive tail curvature, while those with compromised membranes exhibited straight tails. In terms of sperm plasma membrane integrity (Figure 1D), the S1 group demonstrated significantly higher integrity than the S2 group (*p* < 0.01).

(A) Red lowercase ‘a’ indicates acrosome‐intact sperm and ‘b’ indicates acrosome‐damaged sperm. (B) The rate of sperm acrosome integrity. (C) Red lowercase ‘a’ indicates intact plasma membranes sperm and ‘b’ indicates damaged plasma membranes sperm. (D) The rate of sperm plasma membrane integrity. ‘*’ indicates a significant difference (*p* < 0.05) and ‘**’ indicates a statistically significant *p* < 0.01. The same applies below.

### 3.3 Effects of Different Equilibration Methods on Post‐Thaw Sperm Antioxidant Indexes

**FIGURE 2:**
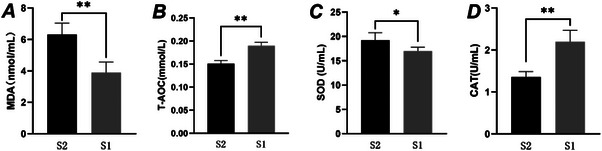
Effects of different equilibrium methods on antioxidant indexes of thawed boar sperm.

Oxidative stress significantly affects sperm quality during the freeze‐thaw process. As shown in Figure 2A, the MDA content in post‐thaw sperm from the S1 group was significantly lower than that in the S2 group (*p* < 0.01). As shown in Figure 2B, the T‐AOC level in the S1 group was significantly higher than that in the S2 group (*p* < 0.01). Additionally, the results in Figure 2D demonstrate that the CAT activity in the S1 group was significantly elevated compared to the S2 group (*p* < 0.01). In contrast, Figure 2C shows that the SOD content in the S1 group was significantly lower than in the S2 group (*p* < 0.05).

### 3.4 Effects of Different Equilibration Methods on Post‐Thaw Sperm Apoptosis Rate

**FIGURE 3:**
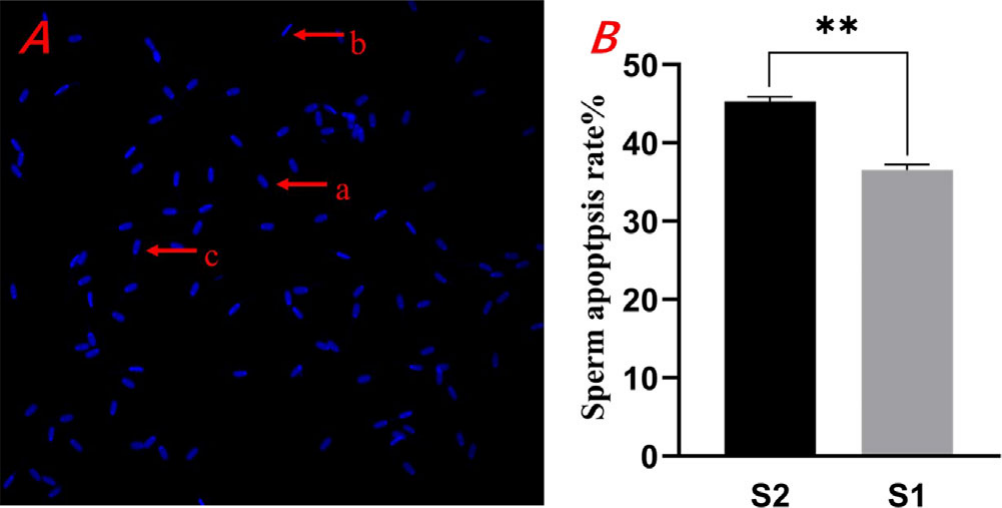
Effect of different equilibration methods on the apoptosis rate of thawed sperm.

The results of post‐thaw sperm apoptosis rates showed that the apoptosis rate in the S1 group was significantly lower than that in the S2 group (*p* < 0.01) (Figure 3B). Under fluorescent microscopy, apoptotic sperm exhibited a bright blue fluorescence, while dead sperm were entirely stained bright blue. In contrast, normal sperm displayed a light blue colour (Figure 3A).

(A) Red lowercase ‘a’ indicates normal sperm, red lowercase ‘b’ indicates dead sperm and red lowercase ‘c’ indicates apoptotic sperm. (B) The sperm apoptosis rate of different equilibration methods.

### 3.5 Effect of Different Equilibration Protocols on the mRNA Expression Levels of Sperm‐Related Genes after Thawing

**FIGURE 4:**
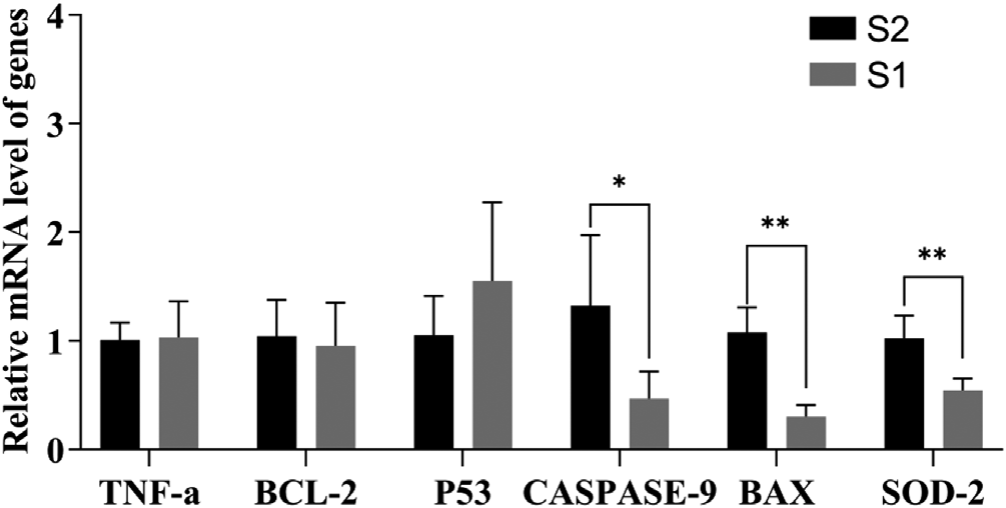
Effect of different equilibration protocols on the mRNA expression levels of sperm‐related genes after thawing.

As shown in Figure 4, the expression level of *Caspase‐9* in Group S1 was significantly lower than that in Group S2 (*p* < 0.05). The expression levels of *Bax* and *SOD‐2* in Group S1 were extremely significantly decreased compared to those in Group S2 (*p* < 0.01). Although the expression levels of *TNF‐α*, *BCL‐2* and *P53* in Group S1 showed some differences compared to Group S2, the differences were not statistically significant (*p* > 0.05).

## 4 Discussion

The equilibration method and duration during the cryopreservation process are closely related to species, freezing protocols and the composition of diluents employed (Nordstoga et al. 2011). However, sperm quality is primarily evaluated in terms of motility, viability and motion parameters. Sperm total motility serves as a key indicator of sperm quality. The study found that S1 produced significantly higher VCL and VAP values than S2 (*p* < 0.05). Although post‐thaw sperm viability, VSL and wobble showed no significant differences between S1 and S2, sperm total motility obtained following S1 cryopreservation was markedly lower than that observed for S2 (*p* < 0.05). These findings are consistent with Monteiro et al. (2022) who reported that sperm total motility after single‐step equilibration was significantly reduced compared to the S2 approach. This decline may be partially ascribed to the shorter equilibration time in S1, which may prevent sperm cells from adequately reaching 4°C prior to exposure to liquid nitrogen vapour. Insufficient acclimatization results in a substantial temperature gradient that induces cold shock, thereby impairing motility and viability (Mazur et al. 1972). Additionally, the cryoprotectant glycerol used in S1 may not have fully penetrated the sperm membrane, reducing the cells tolerance to temperature fluctuations and further compromising post‐thaw motility (Rota et al. 2006).

Cryopreservation processes can diminish membrane fluidity, potentially alter membrane structure and even cause rupture, which negatively impacts sperm functionality and viability (Khan et al. 2021). This study found that the S1 method significantly enhanced sperm plasma membrane integrity following thawing. The plasma membrane is a crucial barrier that plays a fundamental role in the interaction between sperm and egg (Graham and Mocé 2005). Alterations in membrane composition, particularly in the ratio of saturated to unsaturated fatty acids, increase sperm susceptibility to cryodamage and oxidative stress (Park et al. 2023). Lee and Kim (2018) demonstrated that freezing sperm after a 4‐h equilibration at 4°C significantly improved membrane integrity after thawing. In addition to membrane integrity, acrosome integrity is another key indicator of sperm quality. The acrosome reaction during fertilization releases enzymes that help the sperm penetrate the zona pellucida (Karunakaran and Devanathan 2017). This study determined that the acrosome integrity in sperm from the S1 group was higher compared to the S2 group; however, this difference did not reach statistical significance. Previous research indicates that rapid freezing followed by controlled thawing effectively preserves acrosome structure, which is generally consistent with our findings.

Oxidative stress is a critical factor influencing cryopreserved sperm quality. When ROS production surpasses the antioxidant capacity of cells, oxidative damage occurs. Because sperm membranes are rich in polyunsaturated fatty acids, they are particularly vulnerable to ROS‐induced lipid peroxidation, which generates harmful by‐products such as MDA. Elevated MDA levels correlate with reduced motility, abnormal morphology and impaired fertilization capacity (Fu et al. 2024). This study found significantly lower MDA levels in S1 compared to S2 following thawing. Liu et al. (2020) similarly reported increased MDA levels following S2 equilibration. The lower MDA levels observed in S1 may result from the more appropriate equilibration process, which allows sperm to gradually acclimate to lower temperatures, thereby reducing cold shock and subsequent cellular damage. T‐AOC represents the overall antioxidant capacity of sperm cells. Increased T‐AOC is associated with improved total motility, reduced MDA levels and reduced oxidative damage (Li et al. 2023). In this study, T‐AOC was significantly higher in sperm frozen using the S1 method, consistent with earlier findings (Arif et al. 2020). CAT is a critical enzyme that decomposes hydrogen peroxide, thereby protecting cells from oxidative damage. Reduced CAT activity leads to hydrogen peroxide accumulation and impaired total sperm motility and fertility (Wu et al. 2024). This study demonstrated that post‐thaw CAT activity was significantly elevated in S1 group sperm. Prior research indicates that CAT levels decline immediately after thawing but recover during later phases, likely due to antioxidant activation after cryodamage (Buranaamnuay 2017). SOD is another essential antioxidant enzyme, catalysing the conversion of superoxide anions into hydrogen peroxide and alleviating oxidative stress. Reduced SOD activity has been linked to abnormal sperm morphology and impaired motility. This study observed a reduction in SOD levels in S1 group sperm, possibly due to osmotic stress during cryopreservation, which exhausts antioxidant defenses (Buranaamnuay 2017). Research has indicated that SOD activity is influenced by the temperature and duration of thawing, with rapid thawing better preserving SOD activity (Huang et al. 2022).

Apoptosis, or programmed cell death, is another factor influencing sperm quality. Physiological changes during cryopreservation may trigger apoptosis, leading to impaired motility, reduced viability and compromising DNA integrity. The study found that S1 significantly reduced sperm apoptosis following thawing, potentially due to downregulation of pro‐apoptotic genes such as Bax (Hezavehei et al. 2018). Several genes, including TNF‐α, Caspase‐9, Bax and P53, promote apoptosis, while Bcl‐2 inhibits apoptosis, and SOD‐2 acts as an antioxidant (Zhang et al. 2022). Upregulation of Caspase‐9 reduces sperm total motility, hindering their ability to reach the egg (Wei et al. 2015). Our findings, showing reduced Caspase‐9 expression in S1, align with this mechanism (Brugnon et al. 2012). Bax upregulation is known to correlate with poor sperm morphology and motility (Mostafa et al. 2014). Proteins encoded by Bax belong to the Bcl‐2 family and promote apoptosis, whereas Bcl‐2 counteracts apoptosis. SOD‐2 activity is positively associated with sperm concentration and motility, with higher levels reflecting improved sperm quality (Bach et al. 2023). P53‐mediated apoptosis plays a critical role in eliminating damaged cells and maintaining reproductive health. Loss of ARF, a tumour suppressor gene associated with P53, results in increased DNA damage and reduced sperm counts (Zalzali et al. 2018). TNF‐α has been identified as a key mediator of sperm apoptosis, initiating cell death pathways and reducing the number of viable sperm available for fertilization (Mostafa and Taymour 2016). This study found that S1 cryopreservation significantly reduced the expression of Caspase‐9, Bax and SOD‐2, suggesting that multiple apoptotic pathways were attenuated, thereby improving sperm quality. This phenomenon may be attributed to the involvement of multiple apoptotic pathways in mediating sperm cell apoptosis during cryopreservation, which collectively enhance the transmission of apoptotic signals within sperm cells. These findings indicate that S1 is more effective than S2 in mitigating apoptosis and enhancing the quality of cryopreserved sperm.

Although boar studs and AI‐derived semen are not yet widely adopted in East Asian pig production systems—where natural mating remains the predominant breeding strategy—the development of boar studs is gradually increasing as producers seek to improve genetic progress, disease control and biosecurity. To enhance the practical relevance of our findings, we added an analysis comparing the operational costs and benefits of different equilibration protocols from a boar stud perspective. The S1 protocol offers advantages such as reduced handling time, fewer temperature transitions and lower labour requirements, making it more suitable for boar studs with limited technical staff or insufficient cooling facilities. In contrast, the S2 protocol requires more precise monitoring and longer equilibration, increasing operational costs but potentially yielding higher post‐thaw motility. The choice between these protocols in East Asian boar studs therefore depends on balancing labour and cost limitations with the desired post‐thaw semen quality (Monteiro et al. 2022). These findings provide a practical reference for selecting cryopreservation strategies in regions where natural mating remains dominant but AI programmes are expected to expand.

## 5 Conclusion

The experimental results demonstrate differences in post‐thaw sperm quality parameters between the S1 and S2 cryopreservation methods. The S1 method for cryopreserving boar sperm shows superior preservation of membrane integrity, antioxidant levels and apoptosis markers, despite lower vitality and viability. Further investigation of the S1 method is warranted to enhance semen vitality and expand its practical applications.

## Author Contributions

Conceptualization: Biyu Zhang and Fuqiang Chang. Methodology: Haidong Liu and Biyu Zhang. Validation: Biyu Zhang and Fuqiang Chang. Formal analysis: Haidong Liu and Shouqian Sang. Investigation: Biyu Zhang and Jing Li. Data curation: Biyu Zhang. Writing – original draft preparation: Biyu Zhang and Fuqiang Chang. Writing – review and editing: Chongmei Ruan. Visualization: Wenchao Li. Supervision: Chongmei Ruan. Project administration: Biyu Zhang, Chongmei Ruan and Wenchao Li. Funding acquisition: Chongmei Ruan, Youfang Gu and Jing Li. All authors have read and agreed to the published version of the manuscript.

## Funding

This research was funded by the Scientific Research Project of Higher Education Institutions in Anhui Province (grant no. 2023AH051839/2024AH050302), the Scientific Research Foundation of Anhui Science and Technology University for Talent Introduction (DKYJ202005) and Veterinary Science Peak Discipline Project of Anhui Science and Technology University (XK‐XJGF002).

## Disclosure

The statements, opinions and data contained in all publications are solely those of the individual author(s) and contributor(s) and not of MDPI and/or the editor(s). MDPI and/or the editor(s) disclaim responsibility for any injury to people or property resulting from any ideas, methods, instructions or products referred to in the content.

## Ethics Statement

The experimental protocols were approved by the Animal Care Committee of the Anhui Science and Technology University (no. AHSTU2025001). All methods were carried out in accordance with relevant guidelines and regulations. All methods are reported in accordance with ARRIVE guidelines.

## Consent

The authors have nothing to report.

## Conflicts of Interest

The authors declare no conflicts of interests.

## Data Availability

The datasets from this study are not publicly available but are available from the corresponding author on reasonable request.
